# Determination of Metal Content of Waste Mobile Phones and Estimation of Their Recovery Potential in Turkey

**DOI:** 10.3390/ijerph16050887

**Published:** 2019-03-11

**Authors:** Merve Sahan, Mehmet Ali Kucuker, Burak Demirel, Kerstin Kuchta, Andrew Hursthouse

**Affiliations:** 1 Institute of Environmental Sciences, Bogazici University, Bebek, Istanbul 3432, Turkey; mervee.sahan@gmail.com (M.S.); burak.demirel@boun.edu.tr (B.D.); 2 Institute of Environmental Technology and Energy Economics, Waste Resources Management, TUHH—Hamburg University of Technology, Harburger Schloßstr. 36, 21079 Hamburg, Germany; kuchta@tuhh.de; 3 Department of Environmental Engineering, Engineering Faculty, Terzioğlu Campus, Çanakkale Onsekiz Mart University, 17020 Çanakkale, Turkey; 4 Computing Engineering & Physical Sciences, University of the West of Scotland, Paisley PA1 2BE, UK; andrew.hursthouse@uws.ac.uk

**Keywords:** mobile phone, characterization, precious metals, rare earth elements, recovery potential, WEEE

## Abstract

Waste mobile phones constitute one of the fastest growing Waste Electrical and Electronic Equipment (WEEE) types all over the world due to technological innovations and shortening of their life span. They contain a complex mix of various materials, such as basic metals, precious metals and rare earth elements and represent an important secondary raw metal source. The main objectives of this study were to characterize the metal concentration of waste mobile phones by optimizing the inductively coupled plasma optical emission spectrometer (ICP-OES) operation parameters and estimate the metal recovery potential of waste mobile phones in Turkey. Therefore, selected mobile phone samples collected from a recycling center in Turkey were analyzed to determine their metal concentrations. Then, the theoretical recovery potentials of precious and rare earth metals from waste mobile phones were estimated for Turkey. The analytical methods optimized in this study can help further research activities to obtain comprehensive data for determination of the critical metals (precious metals and rare earth elements) in WEEE samples so that proper recycling and recovery strategies can be selected and implemented.

## 1. Introduction

All over the world, the living standards and requirements of societies are altering fast and individuals satisfy their fundamental needs via technology-based items. Therefore, electrical and electronic equipment (EEE) are a significant part of the modern life [1]. Since the digital revolution starting in the 1970s, the rapid pace of technological developments has caused a considerable reduction in the lifespan of most electrical and electronic equipment [2,3]. This period has eventually led to a rapid growth in the amount of unwanted and out of date electronic devices [4,5]. Therefore, a new type of waste has been generated, called Waste Electrical and Electronic Equipment (WEEE) or e-waste, both in the literature and practice [6,7]. The term WEEE is used to define the obsolete forms of all sorts of devices that have parts which transmit and process data by the help of electrical current such as computers, phones, large and small household appliances, lighting equipment or medical devices [8]. In other words, WEEE is defined as electronic devices that are out of date and not functional due to breakdown, physical damage or failure [9,10].

Based on the recent research efforts, it is evident that WEEE generation will be growing very fast in the near future [11]. Most recent studies indicate that the amount of discarded electronic devices that enter the waste stream worldwide is more than 43 million tons annually [12]. Furthermore, it is stated that the amount of e-waste is increasing by 3–5% per year, at a rate that is three times more than that of the municipal solid waste (MSW) raise [13]. Recent studies demonstrate that WEEE makes up 1–5% of the municipal solid wastes, and especially in developed countries, this percentage goes up to 8% [1,14,15,16].

Today, WEEE management is a global problem and it needs international WEEE management solutions [17]. Even though transboundary trade of WEEE is restricted by the Basel Convention, WEEE is still being sent to developing countries, which lack proper regulations regarding public and environmental health [18]. For instance, it has been indicated that the amount of WEEE sent to China increased almost 70% in recent years [19,20]. WEEE seems to be a promising resource to recover precious and rare earth elements, however, if they are not properly managed, basic metals such as Al, Cd, Cr, Pb, etc. can pose significant hazard to the environment and human beings. Eventually, developing countries receiving WEEE are particularly facing serious environmental problems in the management of WEEE [4]. Overcoming these problems seems quite difficult due to the socioeconomic situation of these developing countries [21,22,23]. Therefore, in addition to recovery of valuable materials from WEEE in an economic way, another objective of WEEE management should be rendering it harmless for the environment and human health as well. In mobile phones there are many myriad toxins such as As, Sb, Be, Cd, Cu, Pb, Ni, and Zn. These kinds of chemicals are considered as Persistent Bioaccumulative Toxins (PBTs) and they pose potential risks for the environment when not properly treated.

The WEEE generation rate of countries changes according to the life standard and technological tendencies. High technological equipment usage of Turkey due to its young population causes excess WEEE generation. According to data from the Turkish Ministry of Environment and Urban Planning, the amount of WEEE generated in Turkey is about 539,000 tons per year. In other words, 7 kg of WEEE is generated per person in Turkey [24]. Ozturk stated that 31,510 tons of computers and 2257 tons of mobile phones were discarded in Turkey in 2012 [1]. However, legal WEEE collection rates in Turkey is considerably lower than those of the EU countries. According to the report of the Regional Environmental Center (REC) Turkey, only 1% of generated WEEE was recycled by accredited recycling companies in 2011 [25].

**Figure 1:**
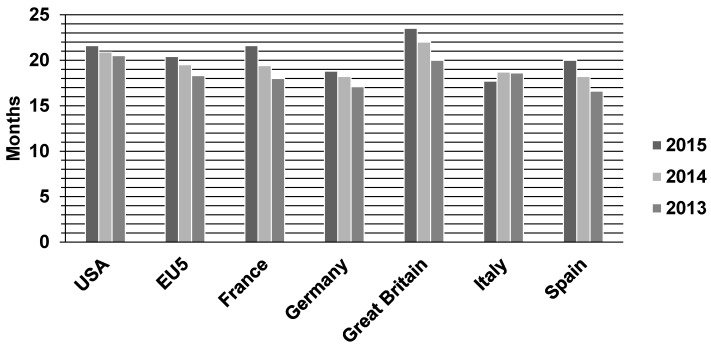
Smartphone life cycle by countries for 2013–2015 [12].

The amount of WEEE has been rising each year due to technological innovations and shortening of lifetimes of electronic equipment. The lifetime of several electronic devices can be estimated as follows: 2–5 years for a computer, 1–2 years for a mobile phone (for smartphones this not more than 18 months to 2 years [12,26,27,28]. The lifecycle of smartphones in selected countries from 2013 to 2015 is shown in Figure 1. Therefore, millions of computers and mobile phones are discarded annually all over the world [29,30].

Table 1 presents the number of waste mobile phones generated in different countries [31,32]. Because of their accumulated amounts and material contents, mobile phones are important secondary sources of valuable materials such as basic metals, precious metals and rare earth elements (REEs) [7,33,34]. Since almost 63% of each WEEE contains valuable and precious metals such as Au, Ag, Cu, Fe, Pb, Al, Hg, Pt, Se, Cd, Cr and Pd, which all have an economic value, recycling and recovery of WEEE has become very attractive around the world today [35,36,37].

Despite its potential economic value, WEEE occupies a large amount of landfill in Nature and the toxic chemicals present in WEEE may pose danger to human health and the environment if not properly managed [15,38]. Therefore, collection and recovery of WEEE has a significant importance for human health and environmental safety [37,39,40,41]. Moreover, since many precious metals and rare earth elements (REEs) are used in the production of electrical and electronic devices, WEEE has a high potential for recovery of valuable elements that can be a great source of raw materials for industrial activities [5,42]. For instance, the annual consumptions of silver and gold in the electronics industry are estimated to be 7554 and 327 tons, respectively [43].

Considering the enormous production of WEEE in recent years, it is obvious that the research of their material composition is essential in order to manage them properly and prevent health and environmental problems resulting from their inappropriate disposal as discussed previously. On the other hand, it is known that the WEEE involves valuable metals as well [2]. Nearly 60% of the WEEE stream consists of seven ferrous and non-ferrous metals such as iron (Fe), copper (Cu), aluminum (Al), lead (Pb), gold (Au), silver (Ag), platinum (Pt) and palladium (Pd). Gramatyka et al. stated that the typical metal scrap comprises 20% copper (Cu), 8% iron (Fe), 4% tin (Sn), 2% nickel (Ni), 2% lead (Pb), 1% zinc (Zn), 0.02% silver (Ag), 0.1% gold (Au) and 0.005% palladium (Pd) metals [44]. Printed circuit boards, popularly known as printed circuit boards (PCBs), are the backbone of most electronics and they are generally composed of metals, ceramics and polymers. Even though they contribute only to 6% of the weight of WEEE, they are the main carriers of valuable metals. According to Cui and Zhang, the precious metal content in telephones and PCBs is about 70%, while it is about 40% in TV boards and DVD players [45]. PCBs of computers and mobile phones contain the highest amounts of valuable metals compared to the PCBs of other electronics, such as televisions, refrigerators, DVD players and calculators [46]. Hageluken stated that a typical computer PCB contains 250 g/ton Au and 20 wt.% Cu, while a mobile phone contains 350 g/ton Au and 13 wt.% Cu [45]. However, the complex structure of WEEE is the main challenge in the recovery of metals from WEEE [47]. Kucuker pointed out that the metals could be recycled by conventional mechanical, pyrometallurgical, hydrometallurgical and bio/hydrometallurgical processes or a combination of these techniques [3]. In addition, the detection and quantification of valuable metals in WEEE samples is crucial for increasing the recycling rate of them. Nevertheless, there exists no standardized or acknowledged method for determining metal content of WEEE. Therefore, information on critical metal content of waste mobile phones and optimum methods for determination of metals that could be employed for WEEE is limited in literature.

The main objectives of this study were to determine elemental concentrations of certain waste mobile phones by optimizing the ICP-OES conditions and to estimate the theoretical recovery potentials from waste mobile phones in Turkey. The metal characterization of fifteen different WEEE samples collected from a recycling center was completed as a first step. Selected basic metals, precious metals and rare earth elements in printed circuit boards (PCBs) and displays of these mobile phone samples were detected and quantified by using optimized ICP-OES conditions. Since the numbers of PCBs of mobile phone samples were relatively higher than those of the number of display samples, their recovery potentials in terms of basic metals, precious metals and rare earth elements were finally determined.

## 2. Materials and Methods

### 2.1. Sample Collection and Preparation

Fifteen (15) different out of use mobile phones were collected from a recycling center in order to determine their metal concentrations and to chemically characterize them. Specifications of waste mobile phone samples used in this study are presented in Table 2. Firstly, samples were disassembled manually and the PCBs and displays of each of them were separated for analyses. For size reduction, separated parts were cut into about 2 × 2 cm pieces using a stainless steel scissor and then crushed into pieces smaller than 2 mm by using a mechanical miller (Retsch SM 300, Haan, Germany). The pieces smaller than 250 μm were sorted by a sieve, collected and then used in the analyses.

### 2.2. Instrumental Analyses

After sample preparation process was accomplished, microwave assisted acid digestion method was employed in order to completely transfer elements into a liquid solution. Nevertheless, a unique digestion method is not available for all solid materials [3]. Therefore, Kucuker evaluated several analytical techniques (EPA Method 3051A, DIN EN 16174, two stage microwave assisted digestion (TSMD)) to decide the optimum conditions for microwave digestion [3]. The heating program of the microwave device was performed in two stages under high pressure according to the digestion method defined by Kucuker [3]. WEEE samples were digested using a Mars 6 Microwave Accelerated Reaction System (CEM Corporation, Matthews, NC, USA) equipped with 12 high pressure 110 mL digestion vessels including a control vessel. 100 mg of each WEEE sample was digested by using 10 mL of HCl (35% m/v) and 3.5 mL of HNO_3_ (69% m/v) acids. All reagents and standard solutions used in this study were of analytical grade. HCl (35% m/v) and HNO_3_ (69% m/v) used during digestion process were purchased from Merck (Darmstadt, Germany). Standard solutions used to construct calibration curves during ICP-OES analyses were prepared from monoelemental high-purity grade 1000 mg/L stock solution of each element (Merck).

In the first stage, the temperature was increased to 140 °C in 15 min. and held at 140 °C for 5 min. In the second stage, the temperature was increased linearly from 140 °C to 200 °C in 16 min. and held at 200 °C for 15 min. The operational conditions for microwave digestion are summarized in Table 3. After microwave digestion program was completed, the vessels were taken out and left for cooling. Then, the digested samples were transferred to clean tubes, diluted to 50 mL with high purity water and filtered from 0.45 µm Syringe Filter. Five different aliquots of each WEEE sample were digested and chemically analysed during this study.

Between each batch of digestion process, the vessels were cleaned by running a cleaning program of microwave digestion system to avoid contamination. After cleaning program was completed, the vessels were filled with diluted HNO_3_ solution and kept until further use. All glassware and polymeric tubes used during the experimental procedure were soaked in a HNO_3_ solution (10% v/v) bath for a day, rinsed with high-purity water and then dried in a clean environment before use.

A simultaneous inductively coupled plasma optical emission spectrometer (ICP-OES, Optima 2100 DV, Perkin Elmer, Waltham, MA, USA) with an axially viewed configuration was used in the study to detect and quantify the selected basic metals, precious metals and rare earth elements. The ICP-OES instrument was equipped with a solid state detector, cyclonic spray chamber and an extended spectral range. High purity grade (99.99%) argon (Ar) gas was used to create plasma during ICP-OES analyses. Standard solutions were used to construct a multipoint calibration curve involving the range of elemental concentrations anticipated in WEEE samples. Standard solutions were prepared from mono-elemental high-purity grade 1000 mg/L stock solution of each element. They were prepared freshly before analysis by diluting with analytical reagent grade HCl (35% m/v) and HNO_3_ (69% m/v) and deionized (DI) water. The selected elements for chemical analyses and the optimized operating conditions of ICP-OES for each element are summarized in Table 4 and Table 5, respectively.

### 2.3. Recovery Potential Method

After the elemental characterization of the WEEE samples was completed, recovery potentials of basic metals, precious metals and rare earth elements were estimated by using the Equation (1) [48]. Recovery potentials of metals were determined by combining elemental concentration values obtained from experimental analyses and certain literature values about WEEE. Prices of metals were determined considering the average prices from reached at the London Metal Exchange [49]. Economic value was calculated by multiplying recovery potential and price of metals obtained from the London Metal Exchange for each metal:

(1) $$\mathrm{Recovery}\;\mathrm{potential}\;\left(\mathrm{tons}/\mathrm{year}\right)\;=\;\frac{\mathrm{element}}{\mathrm{component}\;(\mathrm{PCB})\;}\;\left(\frac{g}{g}\right)\;\times \;\;\frac{\mathrm{component}\;(\mathrm{PCB})}{\mathrm{device}\;(\mathrm{mobile}\;\mathrm{phone})\;}\left(\frac{g}{g}\right)\;\times \;\mathrm{Waste}\;\mathrm{mobile}\;\mathrm{phone}\;\left(\mathrm{tons}/\mathrm{year}\right)$$

Due to the lack of an official information about the amount of discarded mobile phone devices in Turkey, estimated amounts of discarded devices by Ozturk were used to determine recovery potential in this study. Ozturk stated that 2257 tons of mobile phones were discarded in Turkey in year 2012 [1].

In this study, recovery potentials of metals from PCBs of mobile phones were separately determined. The weight percentage of PCBs in mobile phones were generalized as 25% according to information from literature [50,51]. Therefore, these percent weight of PCB as a component in devices were used in the calculations:

(2) $$\frac{\mathrm{metal}}{\mathrm{component}}\;\left(\frac{g}{\mathrm{kg}}\right)=\;\frac{\mathrm{concentration}\;\mathrm{of}\;\mathrm{metal}\;\left(\frac{g}{L}\right)\;\times \;\mathrm{volume}\;\mathrm{of}\;\mathrm{solution}\;(L)}{\;\mathrm{weight}\;\mathrm{of}\;\mathrm{sample}\;(\mathrm{kg})}$$

where volume of solution: 0.05 L; Weight of sample: 0.1 g.

The concentrations of metals in the components of electronic devices were determined by using an ICP-OES in unit of mg/L. Therefore, Equation (2) was used in order to calculate mass fraction of metals in WEEE sample in unit of mg/kg [34,48]. Recovery potentials of basic metals, precious metals and rare earth elements from PCBs of mobile phones were estimated by combining information about metal fractions (g/kg), component percentage (%) and amount of discarded devices (tons) at the end of this study. This is a relative recovery potential approach, since only the extraction rate is considered, omitting the subsequent purification and recovery operations of the process.

## 3. Results and Discussion

### 3.1. Elemental Characterization of Waste Mobile Phones

During this study, thirteen (13) different printed circuit boards and two (2) different displays of several mobile phone samples were chemically analyzed in order to determine their metal concentrations. The results of metal concentrations in PCBs and displays of mobile phone samples are presented separately.

#### 3.1.1. Printed Circuit Boards

Table 6 presents the average concentrations of the detected metals in PCBs of mobile phone samples supplied by a recycling center for this study. The results indicated that copper (Cu) was the metal of the highest concentration in PCBs of each mobile phone sample. The concentration values for copper varied from 206 g/kg (in a Nokia 3210) to 451.4 g/kg (in a Nokia 3410) for all PCB samples. Since copper is one of the most widely used basic metals in electronic devices due to its high conductivity, high copper concentration values in PCB samples were expected.

Iron (Fe) was another basic metal measured in relatively elevated levels in all PCB samples. Fe had a wide concentration range changing between 5 g/kg (in a Nokia 3310) and 48.4 g/kg (in a Nokia 6110). Nickel (Ni) also had high concentration values, ranging from 11 g/kg (in a Nokia 3210) to 59.3 g/kg (in a Nokia 3410). The reason of high nickel concentration in PCB samples might be the use of nickel film under metallic contacts of the keys of mobile phones during manufacturing. However, tin (Sn) and lead (Pb) are the basic metals used in welding of electronic components of PCBs. Their average concentration values in PCB samples were measured from 13 g/kg (in a Siemens C5) to 355 g/kg (in a Nokia 3310) and from 1.0 g/kg (in a General Mobile) to 27.3 g/kg (in a Blackberry smartphone), respectively. The concentrations of chromium (Cr) and cobalt (Co) were detected at relatively low levels in all samples used in this work. Among the precious metals, silver (Ag) and gold (Au) had the highest concentrations in all samples analyzed. While the highest concentration of silver (Ag) was detected to be 8.3 g/kg in a Nokia 3210, the highest concentration of gold (Au) was measured to be 2.9 g/kg in a NG 870. These results may arise from the wide use of silver and gold against oxidation in PCBs [52].

Table 7 shows the minimum, maximum and mean concentration values in weight percent (wt.%) of the thirteen PCB samples evaluated in this study and also the mean concentration values from various studies for comparison of results. Mean concentrations values of copper (Cu), tin (Sn), nickel (Ni), zinc (Zn) and lead (Pb) determined in this study are very similar to the values from studies of Kasper et al. and Yamane et al. [53,54]. However, mean concentration of iron (Fe) is lower than those of the other studies. While the concentration of chromium (Cr) determined by Maragkos et al. is relatively higher than the mean concentration determined in this study, it is still lower than the maximum concentration obtained here [31]. Regarding the precious metals, only silver (Ag) was reported in the study of Yamane et al. and its mean concentration was slightly lower than that of the value reported in this study. Vats and Singh reported that the gold (Au) concentration for 10 different mobile phones ranged from 0.006 to 0.017 (wt.%) [55]. Au values detected in this work are higher. Elemental concentration differences in these studies can arise from variation of brands, models and production date of mobile phones. In addition, analytical methods for chemical characterization and the instruments employed are possibly the other factors that may affect the results from different studies.

#### 3.1.2. Displays

Table 8 shows the average concentrations of the detected metals in displays of two different mobile phone samples supplied by the recycling center. The results indicated that the most abundant metals in displays were silicon (Si), copper (Cu) and aluminum (Al), in descending order. While 21 g/kg Si, 15 g/kg Cu and 12 g/kg Al were detected in Blackberry display samples, the average concentration values of Si, Cu and Al in Nokia display samples were measured as 16.6, 13.5 and 3.8 g/kg, respectively. The reason of high Si and Al concentrations in samples can be the aluminosilicate glass that is commonly used in the displays of mobile phones, which is composed of a mix of alumina (Al_2_O_3_) and silica (SiO_2_).

Silver (Ag) and gold (Au) were the precious metals observed in display samples in low concentrations. Among the rare earth elements (REEs), while lanthanum (La) and cerium (Ce) were detected in both samples, praseodymium (Pr) and dysprosium (Dy) were measured only in Blackberry display. Cerium (Ce) and lanthanum (La) are commonly used rare earth elements in low quantities to produce colors, especially in displays of smartphones. While one of the analyzed display samples was dismantled from an old generation Nokia mobile phone, the other one was the display of a new generation Blackberry smartphone. Therefore, the reason of the concentration differences between two display samples could be the variety of mobile phone models.

### 3.2. Recovery Potential of Waste Mobile Phones

Both the determination of the metal concentrations in single components of WEEE and also the calculation of their recovery potentials is basis of designing effective recycling processes. Therefore, after elemental characterization step was completed, recovery potentials of metals from PCBs of mobile phones were estimated. Table 9 presents the average metal concentrations and recovery potentials of metals from PCBs of mobile phones. Recovery potentials from PCBs of mobile phones were estimated by using the average metal concentration of thirteen (13) different PCB samples determined in the characterization step.

Copper (Cu) has the highest recovery potential from PCBs of mobile phones with 189.1± 11.6 tons per year and its economic value equals to 1.13 million USD. Tin (Sn) and nickel (Ni) have high recovery potentials and they are also two of the most valuable basic metals. Economic values of their recovery are around 0.34 million USD and 0.18 million USD per year, respectively.

PCBs of discarded mobile phones lead to recovery potentials of 2.1 ± 0.3 tons Ag, 0.77 ± 0.1 tons Au, 0.17 ± 0.04 tons Pd and 0.014 ± 0.006 tons Pt per year in Turkey. The total economic value of the recovery of precious metals from PCBs was estimated to be around 37.6 million USD per year.

## 4. Conclusions

The production and manufacturing of electric and electronic equipment is one of the fastest growing industries in the world (growth is estimated at 3–5% per year). Consequently, it is expected that the generation of WEEE will increase globally in near future. It is also very well known that heavy metals found in WEEE may pose significant threat to human health and the environment if they are not properly managed. In addition, WEEE contains appreciable quantities of basic metals, precious metals and rare earth elements with high economic values. However, there is a proportion of valuable metals present in WEEE that is lost in most of current recycling processes despite its high economic value. Therefore, proper management and recycling strategies for handling of WEEE have to be immediately developed and implemented to make use of these resources efficiently. On the other hand, the mobile phones involve many myriad toxins such as arsenic, antimony, beryllium, cadmium, copper, lead, nickel, and zinc which should be properly treated and recovered to diminish the potential risks for air, soil and water sources. Thus, the aim of this study was to determine the recovery potential of metals from waste mobile phones and also to draw attention to the importance of recovery of these metals for the environment.

Although metals are being used with the similar purposes in mobile phones, their concentrations vary according to the structure and model of the electronic products. The results of this study indicate that the concentrations of basic and precious metals in PCBs of mobile phones are higher than those of displays of mobile phones. The total economic value of the recovery of precious metals and of base metals from PCBs was estimated to be around 37.6 and 1.72 million USD per year, respectively. Displays of mobile phones contain REEs, such as lanthanum, cerium, praseodymium and dysprosium. While 0.2 g/kg La and 0.13 g/kg Ce were detected in Blackberry display samples, the average concentration values of La, Ce, Pr and Dy in Nokia display samples were measured as 0.48, 0.004, 0.03 and 0.02 g/kg, respectively. The recovery of precious metals may be the main goal of the recycling process of PCBs from mobile phones, the recovery of REEs should be the focus of the recycling process of displays of mobile phones. In addition, further research activities should incorporate development and establishment of universal analytical methods for rapid and reliable quantification of precious and rare earth elements in different WEEE samples so that appropriate recovery and recycling processes can be selected and applied to benefit from these resources.

## Figures and Tables

**Table 1:** Generation of waste mobile phone in some countries or regions [31,32].

Country	Number of Equipment	Year
USA	130,000,000	Per year
Europe (EU 28)	100,000,000	Per year
China	70,000,000	Per year
Germany	6,500,000	Per year
United Kingdom	18,000,000	Per year
Turkey	22,570,000	2012

**Table 2:** Specifications of WEEE samples used in the study.

Sample No	Category	Model
1	PCB of mobile phone	Nokia 3310
2	PCB of mobile phone	Nokia 6210
3	PCB of mobile phone	Nokia 3210
4	PCB of mobile phone	Siemens C5
5	PCB of mobile phone	Nokia 6110
6	PCB of mobile phone	Nokia 3410
7	PCB of mobile phone	Blackberry smartphone
8	PCB of mobile phone	Mixture of various models (No-name Products—Mix1)
9	PCB of mobile phone	Mixture of various models (No-name Products—Mix2)
10	PCB of mobile phone	Asus Pegasus
11	PCB of mobile phone	General Mobile
12	PCB of mobile phone	NG 870
13	PCB of mobile phone	Nokia C5
14	Display of mobile phone	Blackberry
15	Display of mobile phone	Nokia

**Table 3:** Operational conditions of microwave digestion system.

Operational Parameters	1st Stage	2nd Stage
Power (watts)	800	800
Ramp time (min)	15	16
Temperature (°C)	140	200
Hold Time (min)	5	15
Pressure (psi)	400	600

**Table 4:** Selected elements for chemical analysis of WEEE samples.

Basic Metals	Precious Metals	Rare Earth Elements
Aluminum (Al)	Gold (Au)	Cerium (Ce)
Cadmium (Cd)	Silver (Ag)	Dysprosium (Dy)
Cobalt (Co)	Palladium (Pb)	Lanthanum (La)
Chromium (Cr)	Platinum (Pt)	Neodymium (Nd)
Copper (Cu)		Praseodymium (Pr)
Iron (Fe)		
Lead (Pb)		
Nickel (Ni)		
Zinc (Zn)		
Tin (Sn)		

**Table 5:** ICP-OES operating parameters.

Basic Metals			
Al, Cd, Co, Cr, Cu, Fe, Ni, Pb, Zn		Sn	
Parameter	Value	Parameter	Value
Forward Power	1450 W	Forward Power	1450 W
Plasma gas flow	16 L/min	Plasma gas flow	17 L/min
Auxiliary gas flow	0.6 L/min	Auxiliary gas flow	0.3 L/min
Nebulizer gas flow	0.6 L/min	Nebulizer gas flow	0.6 L/min
Sample uptake rate	1.50 mL/min	Sample uptake rate	1.50 mL/min
Plasma viewing	Axial	Plasma viewing	Axial
Peak algorithm	Peak area	Peak algorithm	Peak area
Measurement point	5 points/peak	Measurement point	7 points/peak
**Precious Metals**			
**Au, Pd, Pt**		**Ag**	
**Parameter**	**Value**	**Parameter**	**Value**
Forward Power	1300 W	Forward Power	1300 W
Plasma gas flow	16 L/min	Plasma gas flow	15 L/min
Auxiliary gas flow	0.6 L/min	Auxiliary gas flow	0.2 L/min
Nebulizer gas flow	0.8 L/min	Nebulizer gas flow	0.8 L/min
Sample uptake rate	2.00 mL/min	Sample uptake rate	1.50 mL/min
Plasma viewing	Axial	Plasma viewing	Axial
Peak algorithm	Peak area	Peak algorithm	Peak area
Measurement point	7 points/peak	Measurement point	5 points/peak
**Rare Earth Elements**			
**Ce, Dy, La, Nd, Pr**			
**Parameter**	**Value**		
Forward Power	1400 W		
Plasma gas flow	15 L/min		
Auxiliary gas flow	1.2 L/min		
Nebulizer gas flow	0.8 L/min		
Sample uptake rate	1.50 mL/min		
Plasma viewing	Axial		
Peak algorithm	Peak area		
Measurement point	7 points/peak		

**Table 6:** Mean concentrations of metals in the PCBs of mobile phone samples.

Element (g/kg Sample)	Asus Pegasus (SN *: 10)	GeneralMobile (SN*: 11)	NG 870 (SN *: 12)	Nokia C5 (SN *: 13)	Nokia 6110 (SN *: 5)	Nokia 3210 (SN *: 3)	Nokia 3310 (SN *: 1)	Nokia 6210 (SN *: 2)	Nokia 3410 (SN *: 6)	Siemens C5 (SN *: 4)	BB Smart ** (SN *: 7)	Mix 1 (SN *: 8)	Mix 2 (SN *: 9)
**Basic Metals**													
Cu	324.7	370.4	227.5	378.0	404.0	206.0	287.5	305.2	451.4	313.1	397.7	282.4	409.8
Fe	23.6	20.4	37.2	33.9	48.4	10.0	5.0	14.8	6.4	11.9	46.3	10.1	34.2
Al	8.9	13.2	10.4	11.5	12.9	10.7	11.8	14.9	16.6	16.3	20.1	15.9	19.7
Sn	62.7	34.3	51.8	28.3	26.2	29.6	35.5	29.7	25.3	13.0	33.0	27.1	13.7
Ni	32.3	13.6	23.8	21.0	37.7	11.0	15.8	31.9	59.3	27.0	17.0	15.0	20.1
Zn	28.1	8.2	21.2	2.3	17.8	3.5	7.2	30.2	67.0	5.1	26.9	13.6	18.9
Cr	0.19	0.11	3.9	0.33	0.51	0.13	0.29	0.46	0.17	0.44	0.44	0.14	15.0
Pb	1.6	1.0	7.3	2.6	16.7	16.3	17.9	14.6	23.3	10.0	27.3	15.6	1.9
Co	0.14	0.05	0.27	0.11	0.19	0.11	0.12	0.37	0.23	0.70	0.05	0.30	0.10
**Precious Metals**													
Ag	2.5	1.7	2.0	2.0	4.7	8.3	5.1	3.7	3.2	3.9	2.6	5.9	1.7
Au	2.4	0.65	2.9	1.4	1.3	1.8	1.6	1.5	0.82	1.1	0.53	1.6	0.17
Pd	0.01	<DL ***	0.04	0.26	0.22	0.36	0.40	0.82	0.12	0.47	<DL ***	0.39	0.14
Pt	0.032	0.022	0.026	0.028	0.050	0.033	0.019	0.007	0.012	0.015	0.026	0.036	0.026

* SN: Sample No (indicated in Table 2), **BB Smart: Blackberry Smart Phone, *** < DL: Below detection limit.

**Table 7:** Metal concentration values (wt. %) in PCBs of mobile phones [3,31,43,44,48].

	Values from This Study				Values from Previous Studies				
Element (wt., %)	Min	Max	Mean	Kasper et al.	Yamane et al.	Maragkos et al.	Xiu et al.	Jing-ying et al.	Kucuker
**Cu**	20.6	45.1	33.5	37.8	34.49	1.77	40.8	39.86	32.62
**Fe**	0.50	4.84	2.32	4.85	10.57	-	0.28	-	1.46
**Al**	0.89	2.01	1.41	0.61	0.26	-	-	-	1.52
**Sn**	1.30	6.27	3.16	2.55	3.39	-	1.6	-	2.37
**Ni**	1.10	5.93	2.50	2.54	2.63	3.02	0.39	0.396	2.93
**Zn**	0.23	6.70	1.92	1.82	5.92	0.10	0.41	0.457	1.70
**Cr**	0.01	1.50	0.17	-	-	0.85	-	-	0.04
**Pb**	0.10	2.73	1.20	1.23	1.87	0.58	1.36	-	1.55
**Co**	0.01	0.07	0.02	-	-	-	-	-	0.02
**Ag**	0.17	0.83	0.36	-	0.21	-	0.106	0.054	0.47
**Au**	0.02	0.29	0.14	-	-	-	0.0065	0.0043	0.14
**Pd**	0.001	0.08	0.03	-	-	-	0.005	-	0.04
**Pt**	0.001	0.005	0.003	-	-	-	-	-	0.002

**Table 8:** Mean concentrations of metals in displays of mobile phone samples.

Element (g/kg Sample)	Blackberry Display (SN *: 14)	Nokia Display (SN *: 15)
**Basic Metals**		
Cu	15.0	13.5
Al	12.0	3.8
Sn	1.2	0.75
Ni	1.4	1.7
Zn	0.30	0.24
Cr	3.7	0.27
Pb	0.26	0.33
Si	20.9	16.6
**Precious Metals**		
Ag	0.22	0.60
Au	0.013	0.19
Pd	<DL **	<DL **
Pt	<DL **	<DL **
**Rare Earth Elements**		
La	0.20	0.48
Ce	0.13	0.004
Pr	<DL **	0.03
Dy	<DL **	0.02
Nd	<DL **	<DL **

* SN: Sample No (indicated in Table 2), ** <DL: Below detection limit.

**Table 9:** Average metal concentrations and recovery potentials of metals from printed circuit boards of mobile phones.

Element	Average Concentration	Recovery Potential	Price of Metals *	Economic Value
	(g/kg)	(tons/year)	($/ton)	(million $/year)
**Basic Metals**				
Cu	335.2 ± 74.0	189.1 ± 11.6	5998	1.134
Fe	23.2 ± 15.2	13.1 ± 2.4	80	0.001
Al	14.1 ± 3.5	7.9 ± 0.6	2079	0.016
Sn	31.6 ± 13.5	17.8 ± 2.1	18,952	0.337
Ni	25.0 ± 13.1	14.1 ± 2.1	12,987	0.183
Zn	19.2 ± 17.3	10.9 ± 2.7	2649	0.029
Cr	1.7 ± 4.1	0.96 ± 0.6	2083	0.002
Pb	12.0 ± 8.7	6.8 ± 1.4	2024	0.014
Co	0.21 ± 0.18	0.12 ± 0.03	25,000	0.003
**Precious Metals**				
Ag	3.6 ± 2.0	2.1 ± 0.3	470,000	0.987
Au	1.4 ± 0.75	0.77 ± 0.1	39,250,000	30.223
Pd	0.29 ± 0.23	0.17 ± 0.04	35,200,000	5.984
Pt	0.03 ± 0.01	0.014 ± 0.002	26,800,000	0.375

* Metal Prices were obtained from the London Metal Exchange on 1 October 2018.
